# NOX4-derived ROS Regulates Aerobic Glycolysis of Breast Cancer through YAP Pathway

**DOI:** 10.7150/jca.81099

**Published:** 2023-08-21

**Authors:** Zhenzhen Zhang, Qiu Luan, Wanting Hao, Yan Cui, Yaming Li, Xuena Li

**Affiliations:** Department of Nuclear Medicine, The First Hospital of China Medical University, Shengyang, China.

**Keywords:** NOX4, breast cancer, reactive oxygen species (ROS), cell proliferation and migration, glycolytic enzyme expression

## Abstract

**Background:** NOX4 is highly expressed in breast cancer and is closely associated with cell invasion and metastasis. The involvement of NOX4 in glycolysis in breast cancer remains unclear. The aim of this study was to investigate the role and mechanism of NOX4 in glycolysis in breast cancer.

**Methods:** NOX4 expression in breast cancer cells was detected by qRT-PCR and western blotting. siRNAs and plasmids were used to silence or enhance the expression of NOX4. The mRNA and protein expression of HK2, GLUT1, PKM2, LDHA, and YAP was detected by qRT-PCR and western blotting, and the ^18^F-FDG uptake rate was detected by γ-radiometer. Detection of reactive oxygen species (ROS) in cells was performed using a commercial ROS kit. After transfection, CCK8, EDU and Transwell experiments were conducted to detect cell proliferation and migration ability. MicroPET imaging was used to detect the effects of NOX4 on tumor metabolism. Immunohistochemistry was used to detect the expression of NOX4, glycolytic enzymes HK2, GLUT1, PKM2, LDHA, the proliferation index KI67, and the activation of YAP pathway molecule.

**Results:** In this study, the expression of NOX4 in MDA-MB-231 and MDA-MB-453 was higher than in MCF10A. qRT-PCR and western blotting experiments showed that NOX4 downregulation decreased the expression of glycolytic enzymes HK2, GLUT1, PKM2, LDHA, and 18F-FDG uptake. Conversely, the overexpression of NOX4 enhanced the expression of HK2, GLUT1, PKM2, LDHA, and 18F-FDG uptake. Proliferation and migration experiments showed that after down-regulation of NOX4, cell proliferation and migration ability decreased, while NOX4 overexpression promoted cell proliferation and migration ability. Additionally, ROS content and YAP expression decreased after NOX4 down-regulation, while ROS content and YAP expression increased following NOX4 overexpression, which was reversed by N-acetyl cysteine (NAC), a ROS inhibitor. Furthermore, exposure to NAC and Peptide17, a YAP inhibitor, blocked the increase in glycolytic enzyme expression, and the enhancement of proliferation and migration caused by NOX4 overexpression. In addition, in animal experiments, the results of the MicroPET imaging showed that the glucose metabolism rate of the NOX4 inhibitor group was significantly lower than that of the control group. ROS levels in the NOX4 inhibitor group was lower than that in the control group. Immunohistochemistry showed that the expression of HK2, GLUT1, PKM2, LDHA, KI67, and YAP in the NOX4 knock-down group were decreased.

**Conclusions:** NOX4 affects breast cancer glycolysis through ROS-induced activation of the YAP pathway, further promoting the proliferation and migration of breast cancer cells.

## Introduction

Cancer statistics in the United States in 2021 show that among women, breast cancer has the highest incidence of cancer and is an important cause of cancer-related deaths 1. Although significant progress has been made in the prevention and treatment of breast cancer in recent decades, due to drug resistance and metastasis, it is urgent to explore the mechanisms that regulate the malignant behavior of breast cancer and to provide novel targets for treatment 2.

Metabolic reprogramming is a key hallmark of cancer. The most frequently studied form of metabolic reprogramming is aerobic glycolysis 3. Compared to normal cells that rely on oxidative phosphorylation as the main energy supply, tumor cells prefer aerobic glycolysis as an energy source, as glycolysis is faster than oxidative phosphorylation, and some biological macromolecules produced by glycolysis can also become raw substrates that participate in tumor proliferation 4. Increased reactive oxygen species (ROS) production and excessive accumulation will lead to changes in the microenvironment of tumors inducing oxidative stress, promoting glycolysis, and metastasis, thus affecting tumor occurrence, angiogenesis, invasion, and metabolism 5. NADPH oxidase, as the main source of ROS, is mainly composed of seven family members: NOX1, NOX2, NOX3, NOX4, NOX5, DUOX1, and DUOX2 6. Compared to other members of the NADPH oxidase family, NOX4 interacts with p22phox without the help of other proteins, and exhibits higher NADPH enzymatic activity and enhanced ROS production 7.

Previous studies on NOX4 have focused on diseases such as atherosclerosis, diabetic vascular disease, and neuropathy 8, 9. In recent years, NOX4 has been found to be of great significance to the occurrence and development of tumors. NOX4 is highly expressed in breast cancer and promotes breast cancer progression in different ways 10. NOX4 promotes the entry of breast cancer cells into the lymphatic vessels and promotes distant metastasis by increasing the germination and formation of lymphatic vessels 11. NOX4 interacts with TGF-β and promotes epithelial-mesenchymal transition and migration through ROS generation 12, 13. NOX4 also affects aerobic glycolysis through multiple pathways. In glioblastoma, ROS produced by NOX4 directly activates the transcription factor FOXM1 through HIF-1α to promote aerobic glycolysis 14. In non-small cell lung cancer, NOX4 induces glycolysis through the ROS/PI3K/AKT pathway, and the NOX4 inhibitor in combination with a glycolysis inhibitor exert a synergistic inhibitory effect on cancer cells 15. However, the impact and mechanism of NOX4 activity in glycolysis of breast cancer cells has not been studied.

The Yes-associated protein (YAP) is the downstream key effector of the Hippo signaling pathway and a carcinogenic factor of many tumors 16 and activation of YAP will increase glycolysis 17. Cho et al. found in hepatocellular carcinoma that YAP expression at the mRNA and protein levels increased after cells were treated with H_2_O_2_, the main component of ROS 18. In gastric cancer, MICAL2 promotes YAP expression through ROS production 19. Therefore, we speculate that NOX4, as the main source of endogenous ROS production and oxidative stress, affects tumor cell glycolysis through the ROS-activated YAP pathway.

## Materials and Methods

### Materials

GKT137831 (NOX4 inhibitor) and Peptide 17 (YAP-TEAD inhibitor1) were purchased from Selleckchem (Houston, TX, USA). N-acetyl cysteine (NAC) was purchased from Sigma Aldrich (St. Louis, MO, USA).

### Cell culture

MDA-MB-231, MDA-MB-453, and MCF-10A cells were purchased from the Shanghai Cell Bank, Chinese Academy of Sciences. MDA-MB-231, MDA-MB-453, and MCF10A cell lines were cultured in DMEM (Hyclone, Logan, UT, USA) medium containing 10% fetal bovine serum (FBS, Clark Bioscience, Houston, Texas, USA) at 37 °C and 5% CO_2_.

### Transfection of NOX4

Breast cancer cells were cultured in six-well plates and transfected when the cells increased to 40%-50% confluence. Using the riboFECT TM CP transfection agent (RiboBio, Guangzhou, China) 100 nM concentration of Si-RNA was transfected according to the manufacturer-s instructions: Si-NOX4 (CCAGGAGATTGTTGGATAA) was used to knock down the expression of NOX4, and Si-NC was used as the negative control. Lipofectamine 3000 (Invitrogen, Grand Island, NY) was used for plasmid transfection to establish the overexpression of NOX4, denoted as NOX4 OE, while the control group transfected with a negative control vector was denoted as NOX4 NC.

### Cell Counting Kit‐8 Analysis

Transfected cells were seeded into 96-well plates (2×10^3^ cells per well) and each sample was seeded in three replicates. After 24 h, 48 h, and 72 h of cell growth, 10 μL Cell Counting Kit‐8 (CCK‐8) (Dojindo, Kumamoto, Japan) reagent was added to each well, and the cells were incubated for 2 h in the cell incubator. The absorbance was detected by a microplate reader (Thermo Fisher Scientific) at a wavelength of 450 nm.

### 5‐Ethynyl‐20‐deoxyuridine Assay

The cells after transfection were counted to prepare 200 μL cell suspension, which was added to each well of a 24-well plate. When the cell density reached 60%-70%, the medium was removed and 5‐Ethynyl‐20‐deoxyuridine (EdU) was added for 2 hours. The cells were then stained and photographed under a microscope.

### Cell Migration Assay

Transfected cells were harvested and a 200 μL suspension containing 4×10^5^ cells was prepared in medium without fetal bovine serum and was added to the upper chamber of the Transwell plate (Corning, NY, USA). A volume of 600 μL of medium containing 20% fetal bovine serum was added to the lower chamber of the Transwell plate. Three replicate wells were established for each condition. Cells were then cultured in a cell incubator for 18 h. After staining with 0.1% crystal violet, cells were observed under a microscope and the images were collected.

### Western Blotting

After transfection, the proteins were extracted with lysate and quantified. Proteins were separated according to molecular weight electrophoresis on a 10% separable gel. After membrane transfer, 5% skim milk was blocked at room temperature for 2 hours, and then incubated with a primary antibody overnight at 4 °C, followed by incubation with a secondary antibody at room temperature for 2 hours. The antibodies and respective concentrations were as follows: NOX4 (1:2000), HK2 (1:1000), Glut1 (1:10000), PKM2 (1:1000), β-actin (1:2000), YAP (1:1000) (Abcam, Cambridge, UK), and LDHA (1:1000) (all from Cell Signaling Technology, Danvers, Mass). Image lab software was used to analyze protein blots obtained.

### Quantitative Reverse‐transcription Polymerase Chain Reaction

Total RNA was extracted with Trizol (Thermo Fisher Scientific, Waltham, MA, USA), and the RNA was reverse transcribed into cDNA using the PrimeScriptTM RT reagent Kit with gDNA Eraser (Takara). SYBR GREEN was used for fluorescence Quantitative Reverse‐transcription Polymerase Chain Reaction (qRT‐PCR) on a Roche 480 Fast Real-Time System (Applied Biosystems, Foster City, CA). qRT-PCR was performed in strict accordance with the kit instructions. The expression of β-actin was used as an internal reference.

### Reactive Oxygen Species Assay

The cell reactive oxygen species detection kit (Biyuntian, Shanghai, China) is based on a DCFH-DA fluorescent probe for ROS detection. Transfected cells were digested and diluted into 1×10^6^ to 2×10^7^ cell suspension in 10 μm DCFH-DA serum-free medium. The cells were incubate in a 37 °C cell incubator for 20 minutes to allow the probe to fully penetrate the cells. ROS generation was detected by flow cytometry (BD, Heidelberg, Germany).

ROS levels in tissue specimens were detected by adding probes (Baiaolaibo, Peking, China) into the homogenate supernatant of the freshly obtained specimens. The homogenates were incubated at 37 °C for 30 min, and then placed in a fluorescence microplate reader (Thermo Fisher Scientific, Waltham, MA, USA). The fluorescence intensity was detected at an excitation wavelength of 488-535 nm and an emission wavelength of 610 nm. The results were normalized according to the corresponding amount of protein.

### Lactic acid Assay

The working fluid was prepared according to the kit instructions (Kaiji, Jiangsu, China), and the lactic acid standard was prepared. The supernatant culture medium of the transfected cells was taken and mixed with the configured working fluid. At the wavelength of 530 nm, the absorbance value was measured and the lactic acid content was calculated.

### ^18^F-FDG Uptake Assay

Cell counting was used to normalized the number of cells in each well consistent. Cell transfection was performed. The cells were cultured in serum-free medium for 2 hours before measurement. Cells were incubated with ^18^F-FDG at a final concentration of 4 μCi/mL for 90 minutes at 37 °C. The radioactivity of each well was measured by γ-counter. The uptake rate was calculated using the formula FDG uptake rate (%) = intracellular CPM count/(intracellular CPM count + supernatant CPM count)×100%.

### Tumor Model and PET Imaging

Female nude mice aged 4-6 weeks were randomly divided into the experimental group and the control group, with five mice in each group. A 200 μL cell suspension was prepared from 1×10^7^ healthy cells and subcutaneously injected into the armpit of the upper limb of each mouse. The mice in the experimental group were injected intravenously with GKT137831 (60 mg/kg per day), and the mice in the control group were injected with the same volume and percentage of DMSO solution. After treatment, mice were scanned by ^18^F-FDG PET (Madick, Shandong, China). Static PET images were collected and ^18^F-FDG (300 uCi/mouse) was injected intravenously on the tail side of the mice. Images were collected 60 minutes after 2% isoflurane anesthesia. After 28 days, the mice were euthanized and the tumors were removed for subsequent experiments. The experimental protocols and procedures used in this study were approved by the Laboratory Animal Department of China Medical University. The animal study was approved by the Ethics Committee of the First Affiliated Hospital of China Medical University.

### Immunohistochemical Analysis

Tumor specimens were obtained and soaked in 10% paraformaldehyde for 48 h to prepare wax blocks for slicing. Immunohistochemical staining (IHC) was performed with the following antibodies: NOX4 (1:250), HK2 (1:500), Glut1 (1:250), PKM2 (1:50), LDHA (1:200), KI67 (1:50), and YAP (1:500) antibody. Images were captured using a microscope.

### Statistical Analysis

GraphPad Prism 7.0 (GraphPad Software, Inc., La Jolla, CA, USA) and SPSS 19.0 (SPSS, Inc., Chicago, IL, USA) were used for statistical analyses. Data were expressed as mean ± standard deviation. An independent sample t test was used for comparison between the two groups. P < 0.05 was considered statistically significant.

## Results

### Expression of NOX4 In Breast Cancer

**Figure 1:**
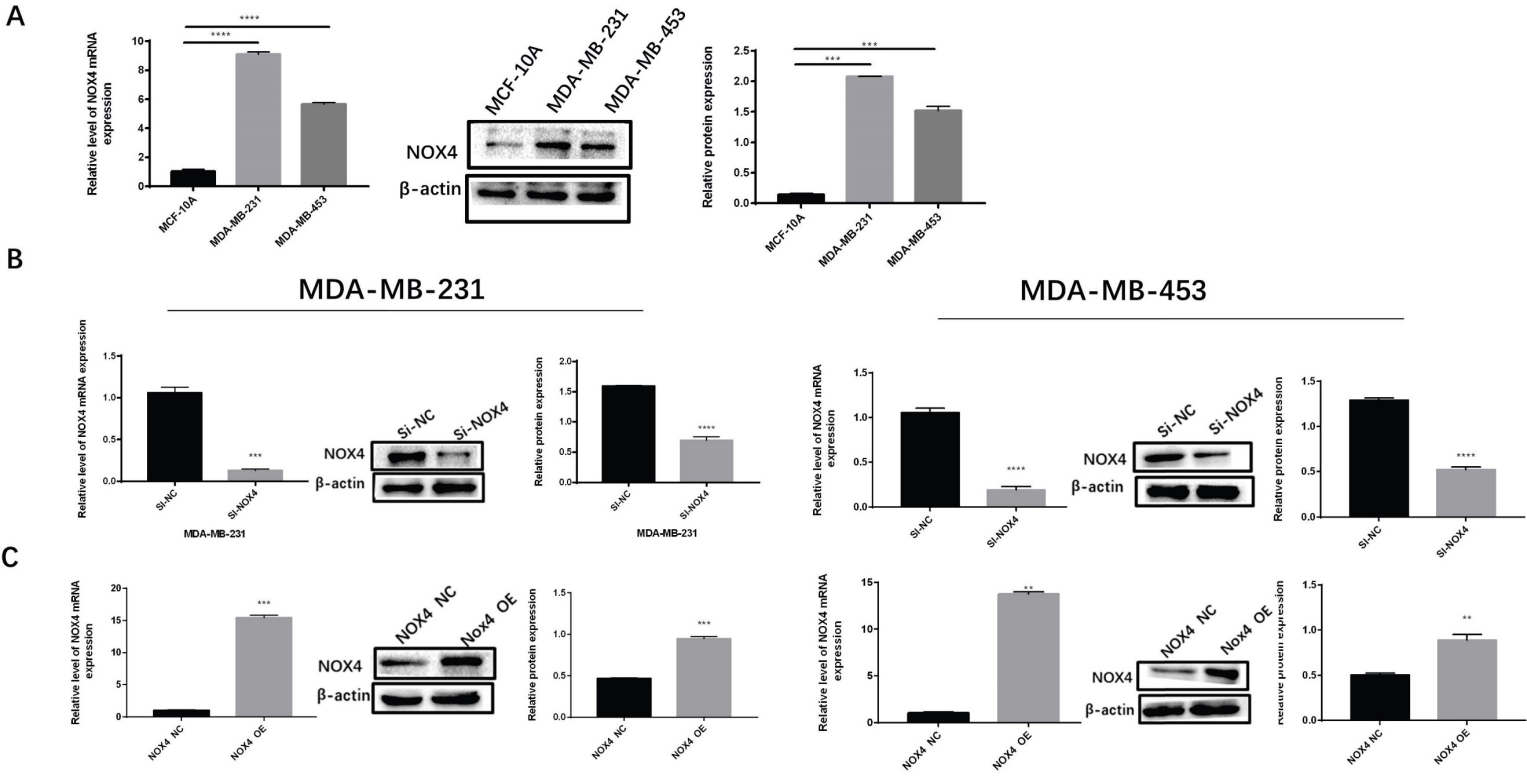
Expression of NOX4 in breast cancer. (A) Expression of NOX4 in breast cancer cell lines (MDA-MB-231, MDA-MB-453) and in the normal breast epithelial cell line MCF10A.(B) Expression of NOX4 in MDA-MB-231 and MDA-MB-453 cells after knockdown of NOX4 by siRNA. (C) Expression of NOX4 in MDA-MB-231 and MDA-MB-453 cells transfected with plasmid. Data represents the mean ± SD, **p < 0.01, ***p < 0.001, ****p < 0.0001.

To investigate the expression of NOX4 in breast cancer, we detected NOX4 expression in MDA-MB-231, MDA-MB-453, and MCF-10A cells by western blotting and qRT-PCR. The results showed that the expression of NOX4 in two breast cancer cell lines MDA-MB-231 and MDA-MB-453 was significantly higher than in normal epithelial cells MCF10A (Figure 1A). Subsequently, we used siRNA to silence NOX4 expression in breast cancer cell lines (Figure 1B), while the NOX4-OE vector was used to over-expression NOX4 levels in breast cancer cell lines for subsequent experiments (Figure 1C).

### Overexpression or Silencing of NOX4 Influenced Breast Cancer Cell Glycolysis

**Figure 2:**
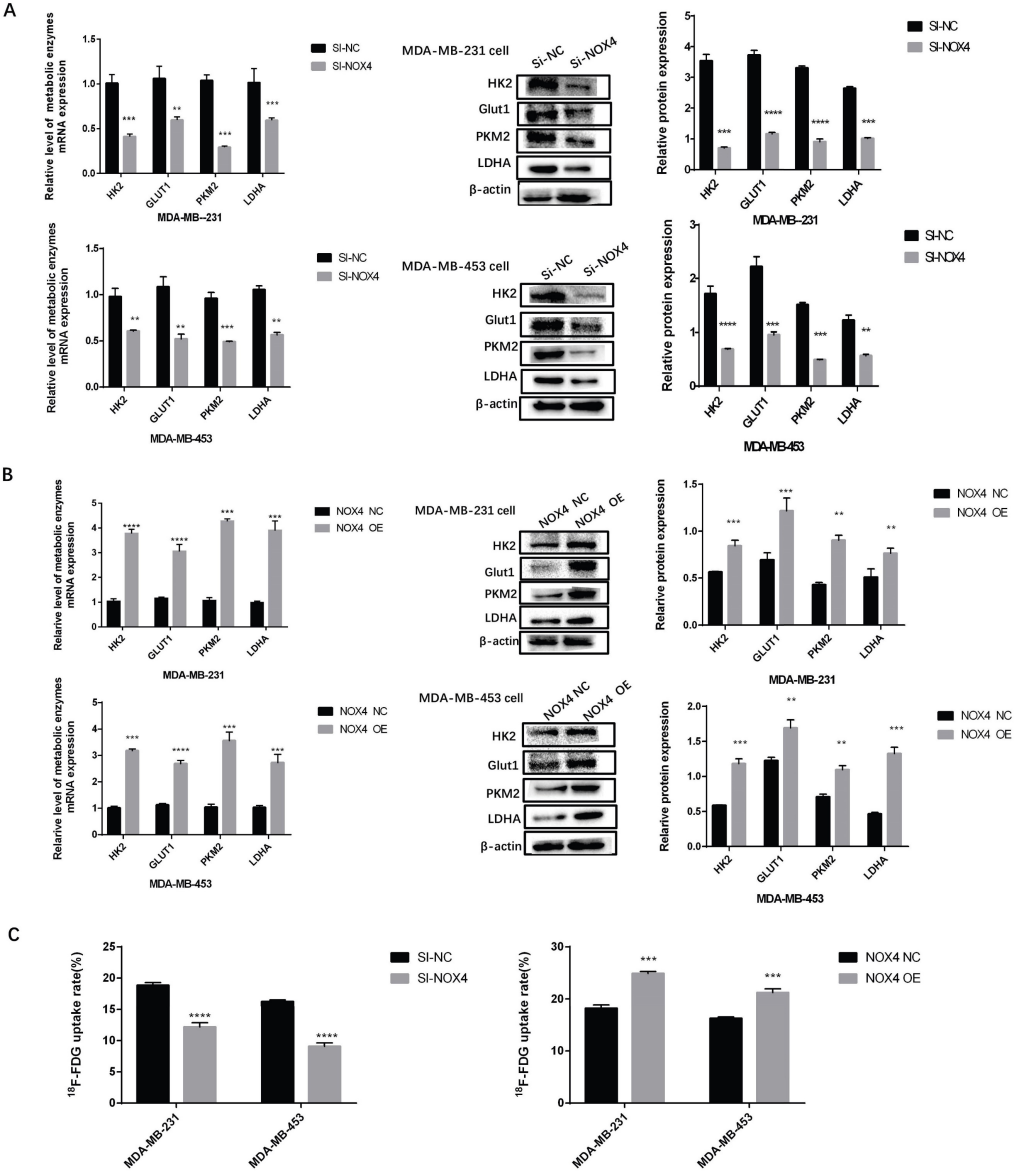
Overexpression or silencing of NOX4 affects tumor cell glycolysis. (A) The expression of glycolysis related enzymes in MDA-MB-231 and MDA-MB-453 cells after NOX4 knockdown. (B) The expression of glycolysis-related enzymes in MDA-MB-231 and MDA-MB-453 cells after increasing NOX4 expression. (C) The ^18^F-FDG uptake rate of MDA-MB-231 and MDA-MB-453 cells after up-regulation and down-regulation of NOX4. Data represents the mean ± SD, *p < 0.05, **p < 0.01, ***p < 0.001, ****p < 0.0001.

To study the effects of NOX4 on the glycolysis of breast cancer cells, qRT-PCR, and Western blot were used to detect key enzymes related to glycolysis after silencing and overexpression of NOX4. Compared to the NC group, the expression of HK2, GLUT1, PKM2, and LDHA in the si-NOX4 group decreased in terms of both RNA and protein levels after knocking down NOX4 expression (Figure 2A). When the expression of NOX4 was enhanced, compared to the NC group, the expression of HK2, GLUT1, PKM2, and LDHA increased at both the mRNA and protein levels (Figure 2B). Lactic acid measurements showed the same trend. The experimental results showed that the silencing of NOX4 reduced the production of lactic acid (Figure S1A, D), the overexpression of NOX4 increased the production of lactic acid (Figure S1B, E). We also used ^18^F-FDG to evaluate the glucose uptake ability of cells. After silencing NOX4, the glucose uptake of cells decreased. In contrast, the NOX4 overexpression resulted in a significant increase in cellular glucose uptake (Figure 2C). The above results show that NOX4 regulates the glycolysis of breast cancer cells.

### Overexpression or Silencing of NOX4 influenced Breast Cancer Cell Proliferation and Migration

**Figure 3:**
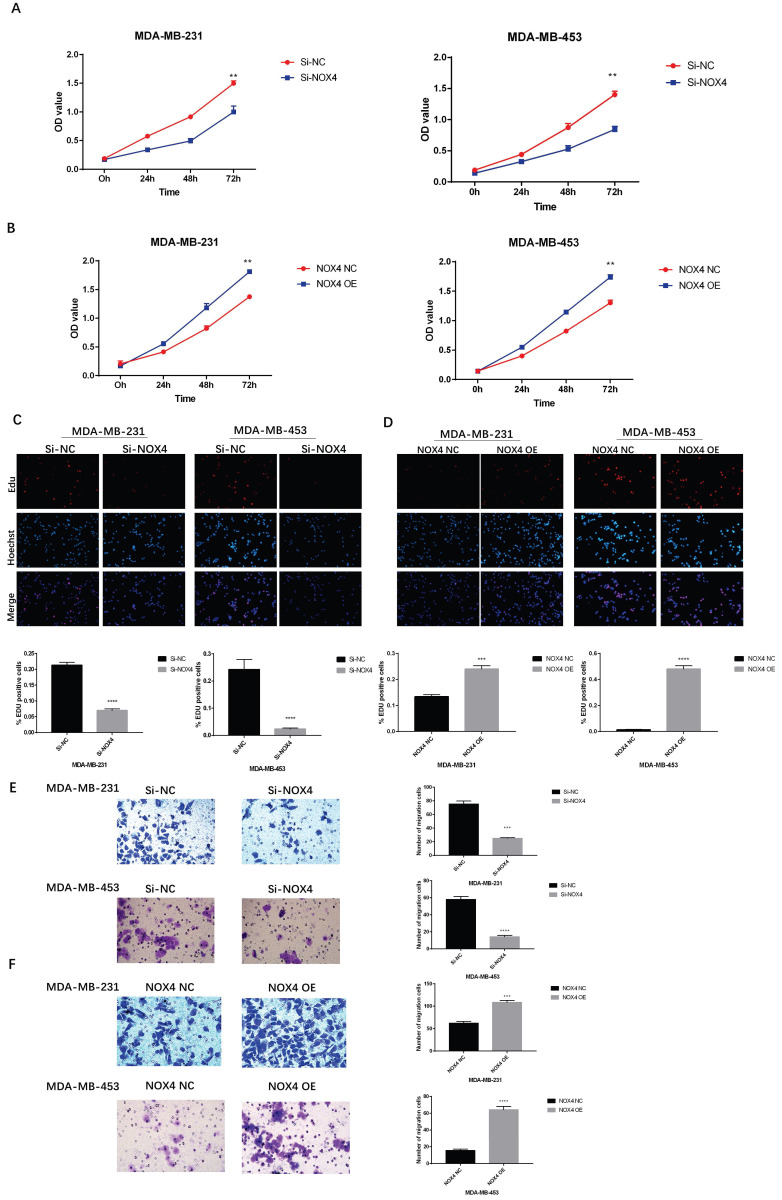
Overexpression or silencing of NOX4 affects tumor cell proliferation and migration (A) The CCK8 assay showing that the proliferation of MDA-MB-231 and MDA-MB-453 cells decreased after the down-regulation of NOX4. (B) The CCK8 assay showing that the proliferation of MDA-MB-231 and MDA-MB-453 cells was enhanced after the up-regulation of NOX4. (C) The EDU assay showing that down-regulation of NOX4 inhibited the proliferation of MDA-MB-231 and MDA-MB-453 cells. (D) The EDU assay showing that the up-regulation of NOX4 enhanced the proliferation of MDA-MB-231 and MDA-MB-453 cells. (E) The results of the invasion experiment showed that the number of migrating MDA-MB-231 and MDA-MB-453 cells decreased after the down-regulation of NOX4. (F) The results of invasion experiment showed that the number of migrating MDA-MB-231 and MDA-MB-453 cells increased after up-regulation of NOX4 expression. The data represent the mean ± SD, *p < 0.05, **p < 0.01, ***p < 0.001, ****p < 0.0001. Magnification 200× (EDU assay); magnification 400× (Transwell assay).

To investigate the effects of NOX4 on breast cancer cell proliferation, CCK8 and EDU experiments were carried out. The results of the CCK8 assay showed that the proliferation of the Si-NOX4 group was significantly inhibited compared to the Si-NC group (Figure 3A). NOX4 overexpression resulted in the opposite result. Compared to the NOX4 NC group, the proliferation of the NOX4 OE group was significantly enhanced (Figure 3B). In addition, we also conducted EDU experiments, which showed the same trend. After down-regulating NOX4 expression, cell proliferation also decreased significantly (Figure 3C); however, after upregulating NOX4 expression, cell proliferation also increased significantly (Figure 3D). Furthermore, we further explored the effects of NOX4 on the ability for tumor cell migration. Down-regulation of NOX4 significantly reduced cell migration (Figure 3E), while up-regulation of NOX4, resulted in a significant improvement of cell migration ability (Figure 3F). In summary, NOX4 is closely related to the proliferation and migration of breast cancer cells.

### NOX4-derived ROS Activated the YAP Signaling Pathways

**Figure 4:**
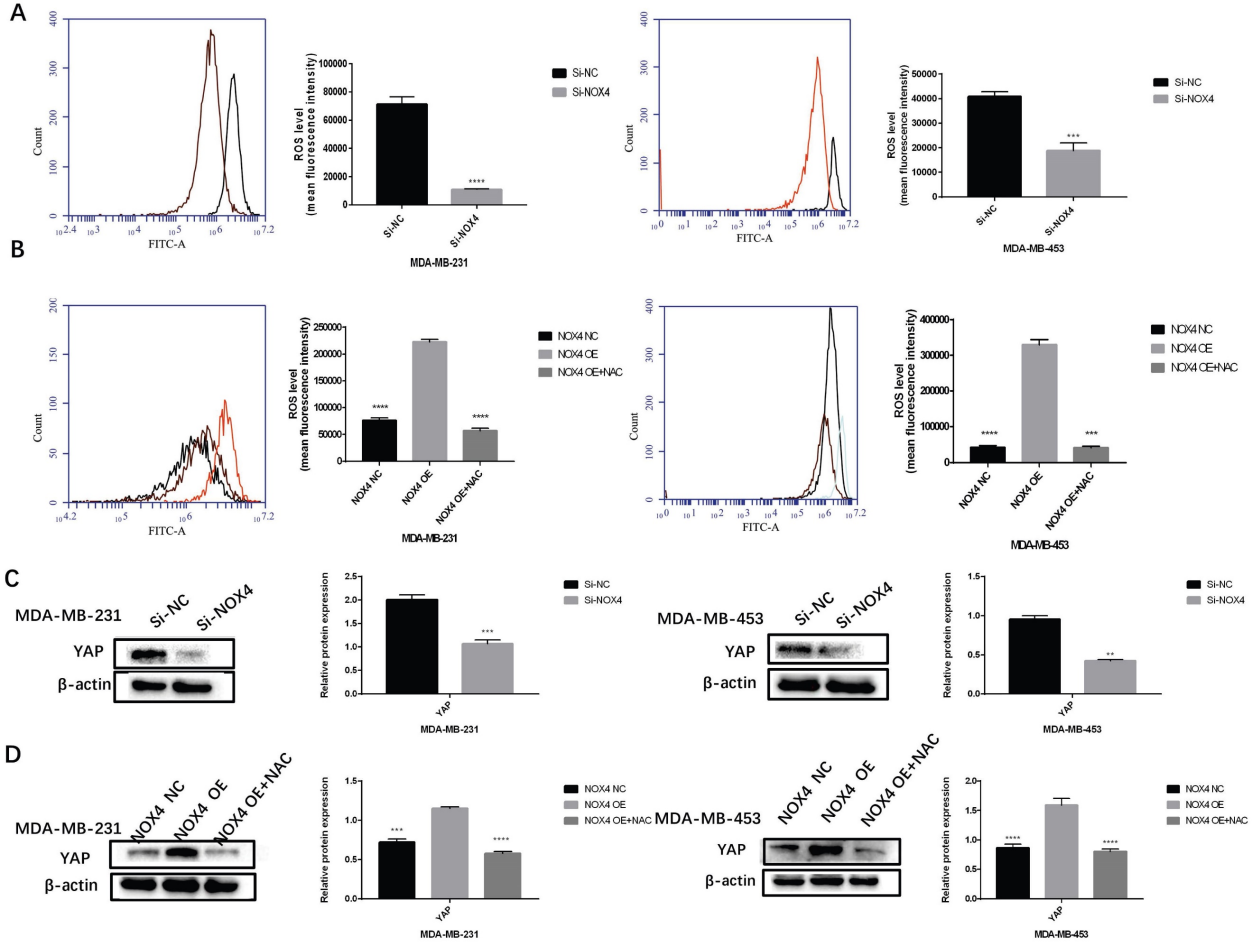
NOX4 derived-ROS activates YAP signaling pathways (A) After down-regulation of NOX4, ROS was detected in MDA-MB-231 and MDA-MB-453 cells. (B) After up-regulation of NOX4, ROS in MDA-MB-231 and MDA-MB-453 cells increased, which was reversed by treatment with N-acetyl cysteine (a ROS scavenger). (C) After NOX4 was down-regulated, the expression of YAP in MDA-MB-231 and MDA-MB-453 cells also decreased. (D) After up-regulating NOX4 expression, the expression of YAP in MDA-MB-231 and MDA-MB-453 cells was increased, which was reversed by exposure to N-acetyl cysteine (ROS scavenger). Data represents the mean ± SD, **p < 0.01, ***p < 0.001, ****p < 0.0001.

To study the mechanism whereby NOX4 regulates glycolysis and transfer, we analyzed the YAP signaling pathway. We verified whether NOX4 activates YAP through ROS. After down-regulating NOX4 expression, ROS production decreased significantly (Figure 4A). Conversely, in cells with NOX4 up-regulation, ROS production was significantly enhanced, which in turn could be blocked by the ROS scavenger NAC (Figure 4B). At the same time, the relationship between NOX4 and YAP was examined. The expression of YAP decreased subsequent to NOX4 down-regulation, but increased after up-regulation of NOX4 (Figure 4C, D). The expression of YAP decreased when cells were exposed to NAC (Figure. 4D). These results showed that NOX4 could activate the ROS/YAP pathway.

### NOX4 influenced Glycolysis, Proliferation and Migration of Breast Cancer Through the ROS/YAP Signaling Pathway

**Figure 5:**
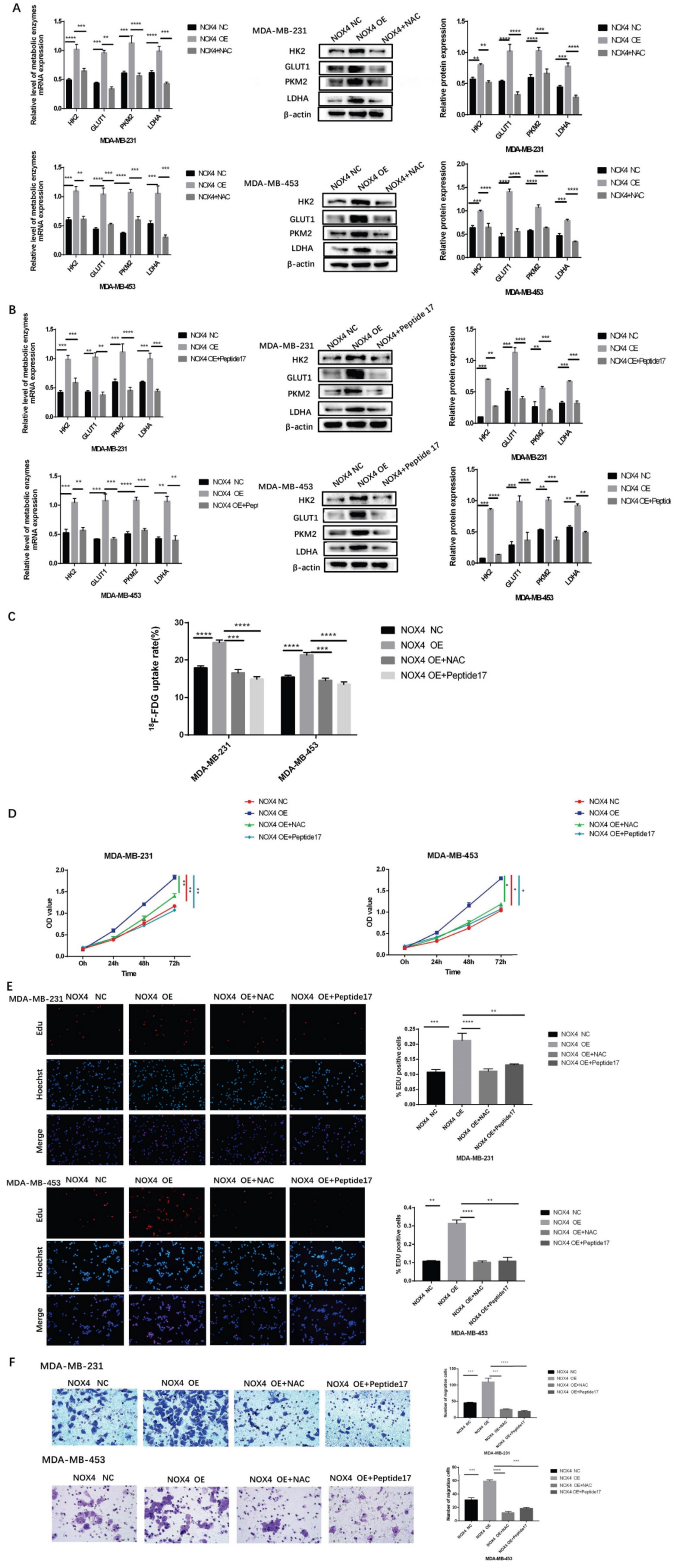
NOX4 affects glycolysis, proliferation, and migration of breast cancer through ROS/YAP signaling pathway. (A) After the application of N-acetyl cysteine (a ROS scavenger), the increase of glycolysis caused by NOX4 overexpression was blocked. (B) Following treatment with Peptide17 (YAP inhibitor), the increase in glycolysis caused by NOX4 overexpression was blocked. (C) The ^18^F-FDG uptake rate of cells was significantly decreased following exposure to N-acetyl cysteine and Peptide17. (D) CCK8 assay results showed that cell proliferation was significantly inhibited after exposure to N-acetyl cysteine and Peptide17. (E) EDU assay results showed that the cell proliferation was significantly reduced after the application of N-acetyl cysteine and Peptide17. (F) The migration experiment results showed that cell migration was significantly reduced after the application of N-acetyl cysteine and Peptide17. Magnification 200× (EDU assay); Magnification 400× (Transwell assay).

We then clarified whether NOX4 influenced the glycolysis and migration of breast cancer through the ROS/YAP pathway. Exposure to NAC and Peptide17 could block the increase of glycolytic enzymes caused by NOX4 overexpression. Compared to the NOX4 OE group, the expression of glycolytic enzymes was significantly reduced in the NOX4 OE cells treated with NAC and in NOX4 OE treated with Peptide17 (Figure 5A and 5B), and the lactic acid produced was also reduced (Figure S1C, F). The ^18^F-FDG uptake assay also revealed the same results. Exposure to NAC and Peptide17 inhibited the overexpression cellular uptake of ^18^F^-^FDG (Figure 5C). The CCK8 assay confirmed that cell proliferation of overexpression cell decreased after treatment with NAC and Peptide17 treatment (Figure 5D). The EDU assay also showed the same results. After NAC (ROS scavenger) and Peptide17 (YAP inhibitor) were applied, overexpressed cell proliferation decreased (Figure 5E). The results of the cell migration experiment also showed that NAC and Peptide17 decreased the ability for overexpressed breast cancer cell migration (Figure 5F). In summary, NOX4 affects glycolysis and proliferation and migration of breast cancer cells through the ROS/YAP pathway.

### Inhibition of NOX4 Reduces Glycolytic Enzyme Expression and Inhibited Tumor Growth *in vivo*

**Figure 6:**
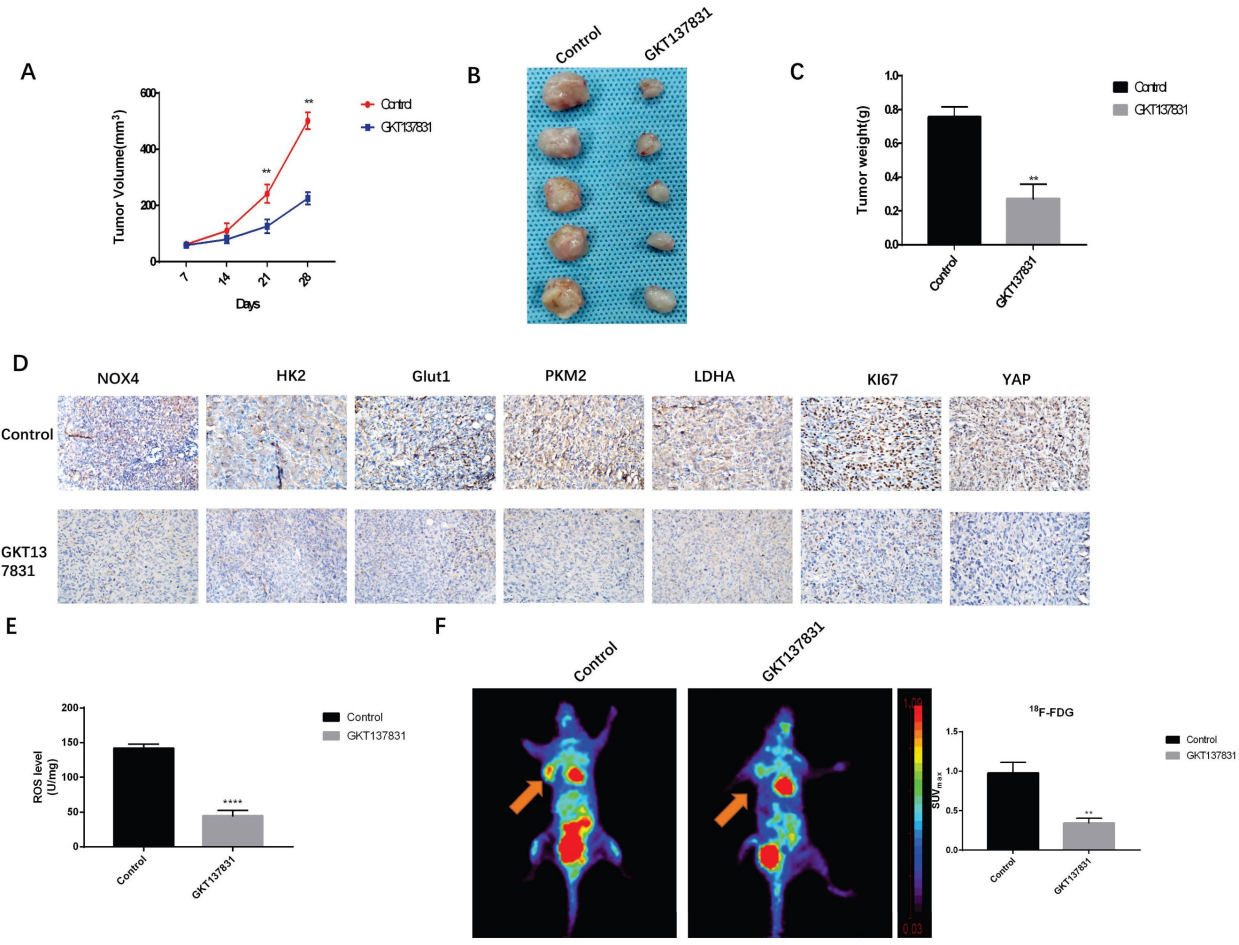
Inhibition of NOX4 reduces glycolytic enzyme expression and inhibits tumor growth *in vivo* (A) GKT137831 inhibits MDA-MB-231 tumor volume compared with the control group. (B) Images of tumor tissues from the Control and GKT137831groups. (C) Compared with the control group, GKT137831 inhibited MDA-MB-231 tumor weight. (D) Immunohistochemical analysis of NOX4, HK2, Glut1, PKM2, LDHA, KI67, and YAP in tumor tissues. (E) Detection of ROS levels in tissues. (F) The SUV_max_ of ^18^F-FDG in the GKT137831 group was significantly decreased. Data represents the mean ± SD, **p < 0.01. Magnification 400×.

To further verify the effects of NOX4 on glycolysis and metastasis of breast cancer cells, we conducted *in vivo* experiments. After planting MDA-MB-231 cells, rats were divided into the NOX4 inhibitor GKT137831 (60 mg/kg daily) treatment group and the control group. The growth of tumor cells was observed. The tumor length and width were measured with a Vernier caliper every 7 days, and the tumor volume was calculated. We observed that GTK137831 could significantly inhibit the increase in tumor volume (Figure 6A). After 28 days, euthanasia was performed on mice and the tumor was resected. Similarly, compared to the control group, the tumor volume and weight of the GKT137831 group were smaller (Figures 6B and 6C). We also found that GKT137831 not only inhibited NOX4 expression, but also inhibited the expression of key enzymes of glycolysis HK2, GLUT1, PKM2, and LDHA, and the expression of the proliferation index KI67 also decreased (Figure 6D), which was statistically significant (Figure S2). The effects of NOX4 on the ROS/YAP pathway was verified and the results showed that GKT137831 significantly decreased the content of ROS and the expression of YAP in tissues (Figure 6D, E). MicroPET imaging was used to further verify the effects of NOX4 on tumor glucose uptake. After the application of GKT137831, the SUVmax value of the tumor decreased significantly (Figure 6F). These results further verified the effect of NOX4 on glycolysis and metastasis of breast cancer.

## Discussion

Aerobic glycolysis is an important indicator of cancer development, which supports tumorigenesis and progression. Targeting tumor glycolysis is also effective for treatment interventions. Many metabolic enzymes have been studied as targets for cancer treatment, such as GLUT1 inhibitor STF-31, HK2 inhibitor phenylnitrobenzylhydrazine, LDHA inhibitor NCI-006D, etc 20-22. Studies have shown that the *in vivo* clinical transformation process of directly targeting inhibition of glucose transporter 1 and hexokinase 1 is slow, and its targeting and metabolic remodeling are key challenges 23. Due to the widespread expression of glycolytic enzymes in organisms and the activation of compensatory pathways, this treatment presents important side effects and uncertain outcomes 24, 25. Therefore, it is essential to explore targets able to regulate tumor metabolism. Previous research gas determined that NOX4 has an effect on tumor metabolism, and a correlation between NOX4 expression and glycolysis can be observed in lung, prostate, and pancreatic cancers 26-28. However, in breast cancer, the effect of NOX4 on glycolysis has not been confirmed.

In this study, we determined that NOX4 expression in MDA-MB-231 and MDA-MB-453 cells was higher than that in the normal breast epithelial cell line MCF10A. Previous studies have shown that NOX4 expression is higher in breast cancer tissues than in paraneoplastic tissues, and NOX4 expression in MDA-MB-231, MDA-MB-435, and BT474 was also higher than in normal epithelial cells MCF12A 29. The expression of glycolytic enzymes was detected after silencing and overexpressing NOX4. After silencing NOX4 expression, the expression of HK2, GLUT1, PKM2, and LDHA, which are key glycolysis enzymes, decreased. Conversely, following the overexpression of NOX4, the expression of these key glycolysis enzymes increased, indicating that NOX4 could regulate glycolysis of breast cancer.

In addition, we investigated whether NOX4 promotes proliferation and migration of breast cancer cells through glycolysis. Cell proliferation and migration were detected after overexpression or silencing of NOX4 expression. The results indicated that silencing of NOX4 inhibited cell proliferation and migration, whereas overexpression of NOX4 promoted cell proliferation and migration. Zhang et al. previously found similar results in the mouse breast cancer cell line 4T1. Silencing NOX4 inhibited the migration of 4T1 cells and reduced the number of pulmonary metastasis nodules and the osteolysis rate in nude mice 13. Furthermore, Ham et al. found that high expression of NOX4 was associated with a reduced survival prognosis of breast cancer patients 30.

We investigated the molecular mechanism of NOX4 regulation of glycolysis. In this study, we first used the public database (GEPIA) to detect the relationship between NOX4 and YAP and found a certain correlation. Subsequently, cell experiments were carried out to verify that ROS production and YAP expression after silencing and overexpression of NOX4. The results showed that ROS and YAP expression decreased after silencing NOX4 expression and increased after NOX4 overexpression. Furthermore, we found that ROS scavenging reversed the increase in ROS and YAP caused by NOX4 overexpression and that inhibition of YAP reversed the increase in YAP expression. These results suggest that YAP is downstream of NOX4-derived ROS.

Furthermore, we explored whether NOX4 could affect the glycolysis and proliferation and migration of breast cancer through the ROS/YAP pathway. Overexpression of NOX4 activated YAP and the glycolysis and proliferation and migration of breast cancer cells were enhanced. Inhibition of ROS and YAP reversed the enhanced glycolysis, proliferation, and migration responses induced by NOX4 overexpression. These results indicated that ROS derived from NOX4 could promote the glycolysis of breast cancer cells through the YAP pathway, thereby affecting cell proliferation and migration. Similarly, it was previously reported that YAP is fully active when cells consume large amounts of glucose activating the glycolytic pathways. Activation of the YAP pathway can promote glycolysis in breast cancer and further promote the proliferation and migration of breast cancer cells 31, 32. When studying the effects of YAP on glycolysis, Zhang et al. found that YAP forms a complex with HIF-1α in the nucleus, binds to the PKM2 gene promoter, and activates its transcription directly 33.

This study also investigated the effects of NOX4 on the glycolysis of breast cancer *in vivo*. In previous studies, it was found that GKT137831 played a significant role in limiting the growth of lung cancer tumors and inhibiting the glycolysis of lung cancer cells by inhibiting NOX4 15. In this study, the tumor growth rate was significantly delayed after the application of the NOX4 inhibitor GKT137831. The immunohistochemical results showed that the expression of NOX4, YAP, and the glycolytic enzymes HK2, GLUT1, PKM2, and LDHA decreased, as did the ROS content. At the same time, the ^18^F-FDG imaging was used for PET scanning to evaluate the effect of the NOX4 inhibitor on aerobic glycolysis in breast cancer. ^18^F-FDG is a radiolabeled glucose analogue. Due to the Warburg effect, tumor cells will increase the uptake of glucose. An abnormal accumulation of 18F-FDG was revealed in the PET scan images. The experimental results showed that the SUVmax of the GKT137831 group was significantly lower than that of the control group. microPET imaging *in vivo* reflects increase in glucose transport. All of the above data further indicated that NOX4 could regulate breast cancer glycolysis through the ROS/YAP pathway.

Based on the analysis of the experimental results above combined with the auxiliary verification MicroPET imaging findings in animal experiments, this study demonstrates that NOX4 affects the aerobic glycolysis of breast cancer. NOX4 activates the YAP pathway through ROS, which in turn influences breast cancer glycolysis, further promoting breast cancer proliferation and migration.

## Conclusion

NOX4 affects breast cancer glycolysis through ROS-induced activation of the YAP pathway, further promoting the proliferation and migration of breast cancer cells.

## Supplementary Material

Supplementary figures.

## Abbreviations

NOX4: NADPH oxidase 4
NADPH: Reduced form of nicotinamide adenine dinucleotide phosphate
ROS: reactive oxygen species
HK2: Hexokinase 2
GLUT1: Glucose transporter protein-1
PKM2: Pyruvate kinase isozymes M2
LDHA: Lactate dehydrogenase A
YAP: Yes-associated Protein
