# Supplementary material for: NOX4-derived ROS Regulates Aerobic Glycolysis of Breast Cancer through YAP Pathway
Source: J Cancer. 2023 Aug 21;14(13):2562–73. doi: 10.7150/jca.81099 (PMC10475359; doi:10.7150/jca.81099)
Supplement: Supplementary file 1 — Supplementary figures. [file jcav14p2562s1.pdf]

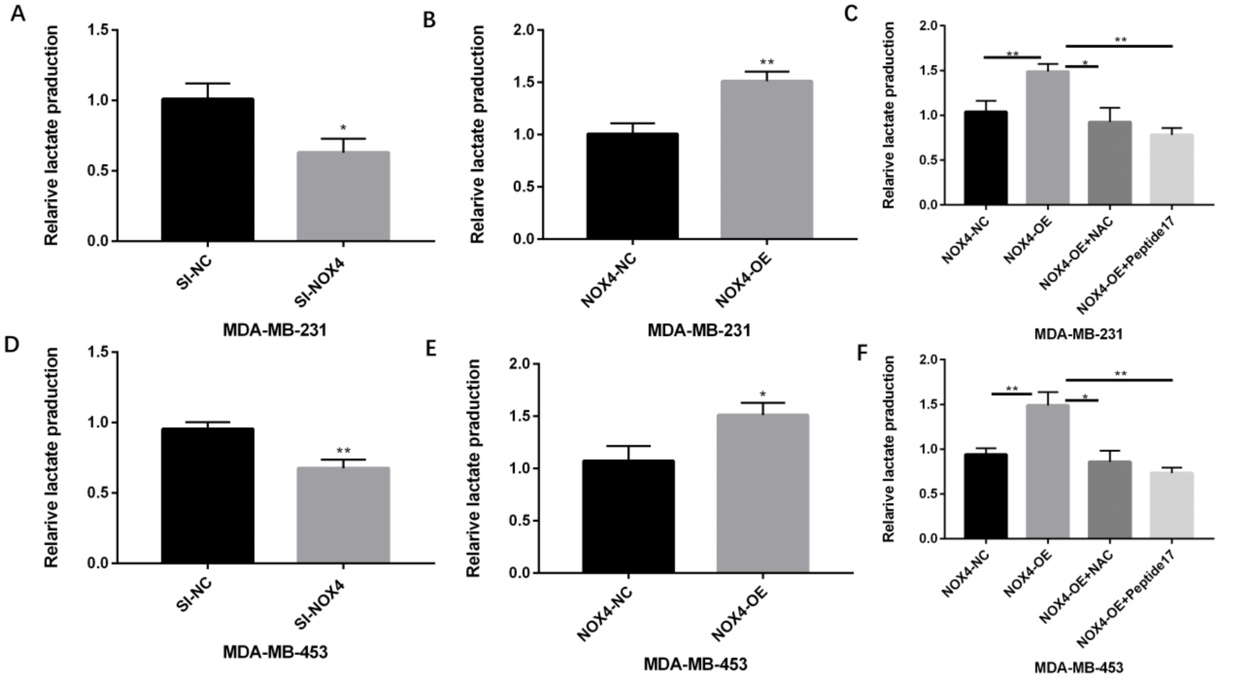


**Figure S1.** Experimental results of lactic acid determination (A) In MDA-MB-231 cells, NOX4 knockdown reduced lactate production. (B) In MDA-MB-231 cells, overexpression of NOX4 increased lactate production. (C) In MDA-MB-231 cells, after application of ROS and Peptide17, the production of lactic acid in overexpressed cells decreased. (D) In MDA-MB-453 cells, NOX4 knockdown reduced lactate production. (E) In MDA-MB-453cells, overexpression of NOX4 increased lactate production. (F) In MDA-MB-453 cells, after application of ROS and Peptide17, the production of lactic acid in overexpressed cells decreased. Data represents the mean ± SD, *p < 0.05, **p < 0.01.

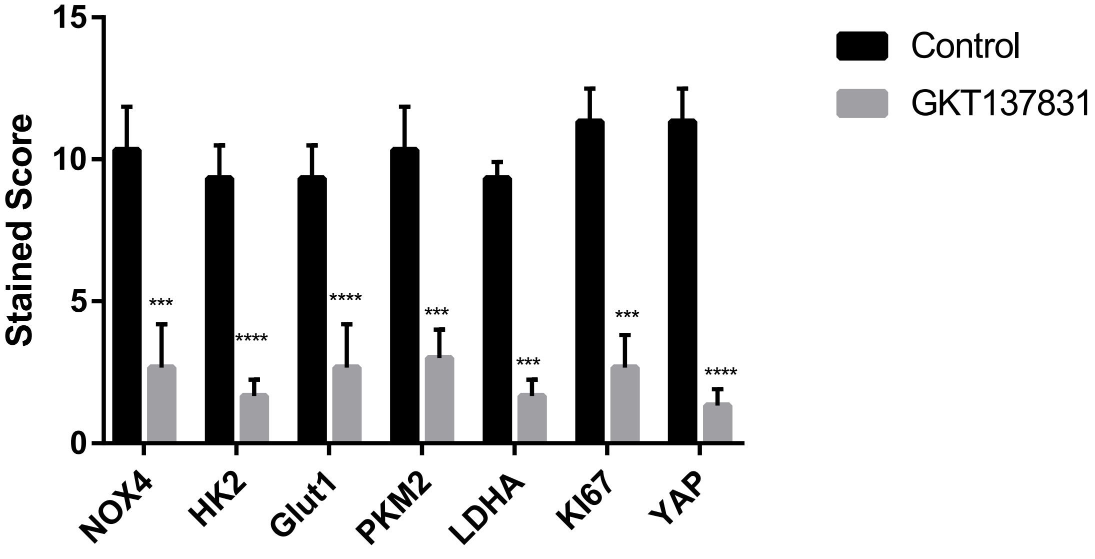


**Figure S2.** Results of statistical analysis of immunohistochemical data. Data represents the mean ± SD, ***p < 0.001, ****p < 0.0001.
